# Cholinergic Crisis Associated With Standard-Dose Pyridostigmine in a Hemodialysis Patient With Ocular Myasthenia Gravis and Marked Weight Loss

**DOI:** 10.7759/cureus.104810

**Published:** 2026-03-07

**Authors:** Kinoshita Junki, Yusuke Nozaki, Kentaro Miyake

**Affiliations:** 1 Anesthesiology and Critical Care, Kasugai Municipal Hospital, Kasugai, JPN

**Keywords:** aspiration, cholinergic crisis, continuous hemodiafiltration, frailty, hemodialysis, hypercapnic respiratory failure, japanese geriatrics, myasthenia gravis, pleural effusion, pyridostigmine

## Abstract

Pyridostigmine is an acetylcholinesterase inhibitor widely used for the symptomatic treatment of myasthenia gravis (MG). Cholinergic crisis is a rare but life-threatening adverse effect caused by excessive cholinergic stimulation. Since pyridostigmine is primarily excreted by the kidneys, standard doses may become excessive in the presence of severe renal impairment. We report a case of cholinergic crisis in an elderly patient on maintenance hemodialysis (HD) who exhibited significant weight loss and severe hypoalbuminemia. An 81-year-old male with acetylcholine receptor antibody-positive ocular MG on maintenance HD developed acute loss of consciousness and severe hypoxemia immediately after his usual pyridostigmine therapy (60 mg twice daily). He presented with marked miosis, profuse secretions, severe bradycardia, and hypotension. Arterial blood gas analysis revealed extreme hypercapnia, and serum cholinesterase (ChE) levels were markedly depressed (17 U/L; reference range: 240-486 U/L). Pyridostigmine was immediately discontinued, and atropine was administered via bolus followed by continuous infusion. During the period of hemodynamic instability, the patient required tracheal intubation and continuous hemodiafiltration (CHDF). Continuous atropine administration was gradually tapered and discontinued on day 12. A tracheostomy was performed on day 12, the patient was successfully weaned from mechanical ventilation on day 16, and he was discharged from the intensive care unit (ICU) on day 22. This case underscores that even standard doses of pyridostigmine can induce a cholinergic crisis in patients with severe renal failure receiving HD. Furthermore, significant weight loss and severe hypoalbuminemia may have increased the patient's vulnerability to severe cholinergic toxicity.

## Introduction

Pyridostigmine, an acetylcholinesterase inhibitor, is widely used as the first-line symptomatic treatment for myasthenia gravis (MG) [1,2]. Cholinergic crisis, caused by excessive cholinergic stimulation, is rare but can be fatal due to respiratory failure or circulatory collapse [3]. Pyridostigmine is primarily excreted by the kidneys; in cases of severe renal impairment, its elimination is prolonged, and systemic clearance is reduced. Therefore, there is a concern that standard doses may be excessive for patients with advanced renal dysfunction, including those on hemodialysis [4,5]. Classic pharmacokinetic data show that severe loss of renal function markedly prolongs pyridostigmine elimination (the elimination half-life increasing by approximately threefold) and reduces plasma clearance several-fold [4]. Therefore, even patients with clinically stable ocular MG on long-term dosing may become vulnerable to toxicity when renal function changes or when physiological reserve declines.

Frailty-related characteristics, such as significant weight loss and hypoalbuminemia, may reflect a decline in physiological reserve in elderly individuals [6,7]. We report a case of cholinergic crisis following the administration of a standard dose of pyridostigmine in an elderly patient with ocular MG on maintenance hemodialysis, against a background of functional decline following a fracture, marked weight loss, and severe hypoalbuminemia.

The contents of this paper were presented as a poster at the Congress of the Japanese Society of Intensive Care Medicine in March 2025.

## Case presentation

An 81-year-old male was diagnosed with MG approximately 20 years ago (details unknown) and followed as AChR antibody-positive, anti-MuSK antibody-negative ocular MG. He had a history of thymectomy and had been maintained on 60 mg of pyridostigmine twice daily for a long period. There had been no recent exacerbation of MG symptoms or changes in dosage. Meanwhile, maintenance hemodialysis (three times weekly, four hours per session) had been initiated five years prior due to nephrosclerosis. The pyridostigmine dosage remained unchanged following the initiation of dialysis.

Approximately four months before admission, the patient suffered a femoral fracture, which led to a decline in activities of daily living (ADL). During this period, his body weight decreased from 64 kg to 53.5 kg (−16.4%). His height was 162 cm, and his BMI had decreased from 24.4 kg/m² to 20.4 kg/m². Outpatient medications included furosemide, aspirin, warfarin, and amlodipine.

The patient presented with acute hypoxemia and transient loss of consciousness several hours after his last dose of pyridostigmine. Upon arrival, he exhibited severe hypotension (61/36 mmHg) and bradycardia (30 bpm). Physical examination revealed marked miosis, profuse secretions, lacrimation, and diaphoresis. Arterial blood gas analysis showed extreme hypercapnia (pH 6.790, PaCO2 164.6 mmHg, PaO2 29 mmHg, HCO3− 24.5 mmol/L, lactate 1.59 mmol/L). Serum cholinesterase was 17 U/L (reference range: 240-486 U/L) and further decreased to a nadir of 11 U/L on day 3. Serum potassium was 3.5 mEq/L and creatinine was 4.47 mg/dL. The complete laboratory findings, including reference ranges, are summarized in Table 1. There was no clinical evidence suggesting an acute exacerbation of MG (myasthenic crisis). 

**Table 1:** Laboratory findings at presentation and during hospitalization.

Parameter	Unit	Reference range	Day 1
Cholinesterase	U/L	240–486	17
Albumin	g/dL	3.8–5.3	2.7
BUN	mg/dL	8–20	29.2
Creatinine	mg/dL	0.65–1.07	4.47
Potassium	mEq/L	3.6–4.8	3.5
Lactate	mmol/L	0.5–2.0	1.59
pH	-	7.35–7.45	6.79
PaCO2	mmHg	35–45	164.6
PaO2	mmHg	80–100	29
HCO3-	mmol/L	22–26	24.5

An atropine bolus of 0.5 mg was administered, followed by continuous intravenous infusion starting several hours after ICU admission. The continuous dose began at 10 mg/day and was gradually tapered and terminated by day 12. Due to coma and respiratory/circulatory failure, the patient was intubated on day 1. Although he was extubated on day 2 following clinical improvement, re-intubation was required on day 5 due to an increased respiratory rate (24 breaths/min), hypercapnia (PaCO2 58.5 mmHg), frequent suctioning needs, and atelectasis. A tracheostomy was performed on day 12, the patient was successfully weaned from mechanical ventilation on day 16, and he was discharged from the ICU on day 22.

**Figure 1:**
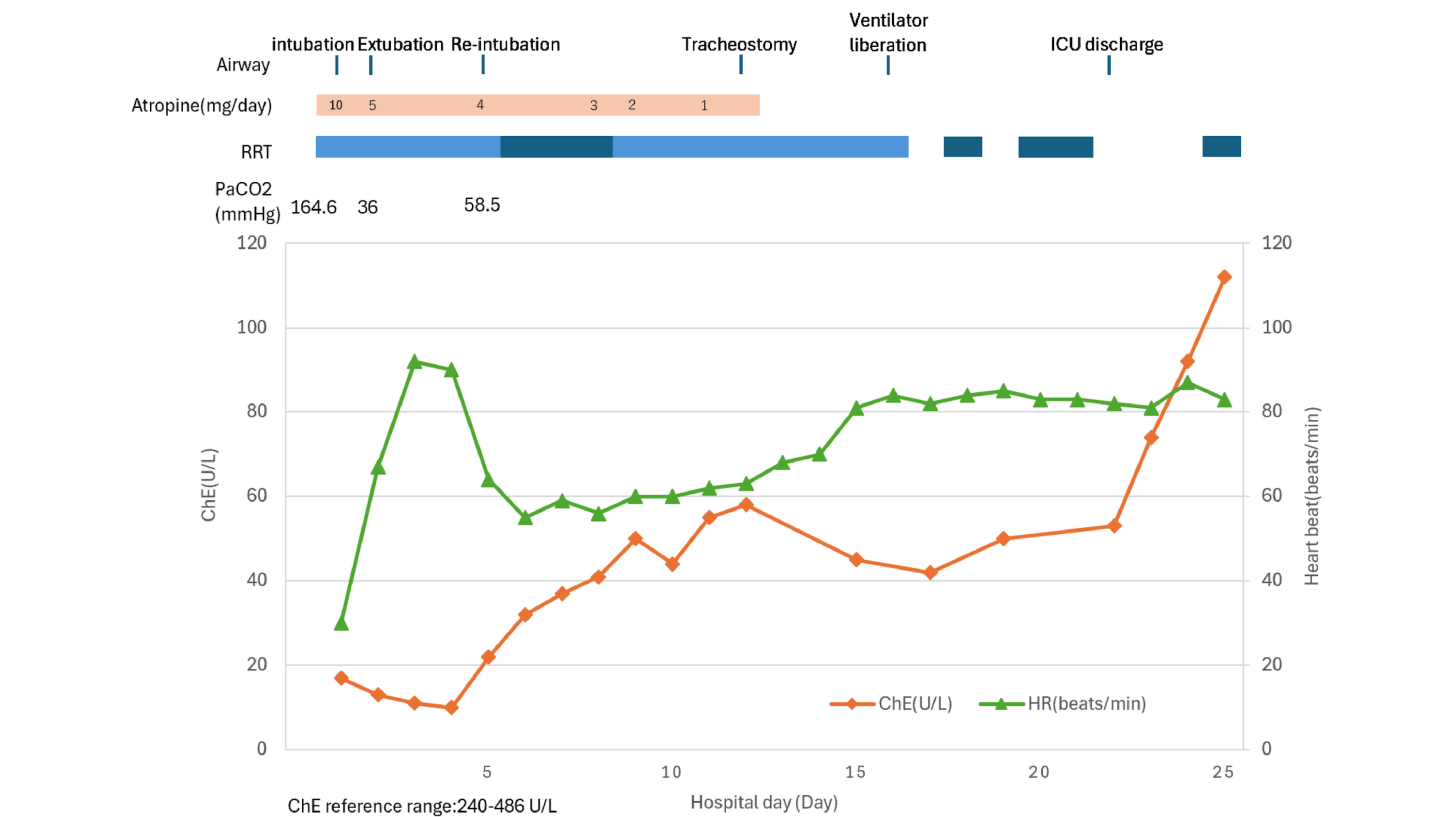
Clinical course during the ICU stay (days 1–25). Abbreviations: ICU, intensive care unit; ChE, cholinesterase; PaCO2, arterial partial pressure of carbon dioxide; RRT, renal replacement therapy; CHDF, continuous hemodiafiltration; HD, hemodialysis Image created by the authors with MS Excel (Microsoft Corp., USA)

Renal replacement therapy during the ICU stay consisted of a combination of continuous hemodiafiltration (days 1-5, 9-16) and intermittent hemodialysis as appropriate. Serum albumin was 2.7 g/dL on day 1 and decreased to a nadir of 1.9 g/dL between days 16 and 22. The detailed clinical course, including serial laboratory trends, ventilatory management, and renal replacement therapy, is summarized in Figure 1.

## Discussion

This report highlights two critical points. First, in patients with advanced renal impairment receiving maintenance hemodialysis, even standard doses of pyridostigmine can induce a life-threatening cholinergic crisis. Second, frailty-related vulnerability, suggested by functional decline following a fracture, significant weight loss, and severe hypoalbuminemia, may have lowered the threshold for severe cholinergic toxicity in this elderly patient.

While pyridostigmine is widely prescribed for the management of MG symptoms [1,2], its excretion is highly dependent on renal function. Pharmacokinetic data and prescribing information indicate prolonged elimination and reduced clearance in severe renal impairment, supporting dose reduction and close monitoring [4,5]. Although reports of pyridostigmine-induced cholinergic crisis specifically in the context of renal failure are limited, cases occurring during acute deterioration of renal function have been reported, demonstrating the importance of re-evaluation during renal impairment [8]. In contrast, cholinergic crises induced by distigmine bromide, a longer-acting acetylcholinesterase inhibitor, have been repeatedly reported, especially in elderly and renal failure patients [9-12]. This case suggests that the clinical lessons learned from distigmine should also be applied to pyridostigmine: in the presence of advanced renal failure, even “standard” doses can become excessive.

From a pharmacokinetic perspective, pyridostigmine elimination is highly dependent on renal function. In classic data comparing subjects with normal renal function and anephric patients, the elimination half-life increased from approximately 112 minutes to 379 minutes, and plasma clearance decreased from about 9 mL/kg/min to 2 mL/kg/min, indicating a substantial renal contribution to overall clearance [4]. Product labeling, therefore, recommends careful dose selection and titration in renal impairment because the risk of toxicity may be increased [5]. In addition, renal clearance studies and pharmacokinetic summaries describe prominent renal elimination with minimal plasma protein binding and a moderate volume of distribution, which supports the plausibility of accumulation when renal function is severely impaired [13,14]. The dialyzability of pyridostigmine during intermittent hemodialysis or CRRT has not been well characterized; commonly used dialysis drug references list pyridostigmine as “ND” (no data) for dialyzability [15]. In general, extracorporeal removal during CRRT depends on drug properties (e.g., protein binding, volume of distribution, molecular size) and the delivered CRRT dose, with effluent rate being a key determinant of solute/drug clearance [16]. Accordingly, although renal replacement therapy was used in this case during hemodynamic instability and with the intent of facilitating drug removal, drug concentrations were not measured, and the contribution of extracorporeal clearance cannot be determined.

Host-side vulnerability is also thought to have contributed to the severity. The patient exhibited significant weight loss, low BMI, and hypoalbuminemia following functional decline after his fracture. These features of frailty in the elderly reflect reduced physiological reserve and vulnerability to acute decompensation [6,7]. Although evidence directly linking frailty to pyridostigmine toxicity is limited, reduced respiratory muscle reserve and impaired clearance of secretions may increase the risk of respiratory failure once a cholinergic state occurs. Case reports of distigmine also describe similar severe respiratory and circulatory failure in elderly or debilitated patients, supporting the concept that host factors amplify toxicity [9,10].

The diagnosis of cholinergic crisis was supported by prominent muscarinic signs (miosis, profuse secretions, diaphoresis), severe bradycardia and hypotension, extreme hypercapnia, markedly low serum cholinesterase levels, the temporal relationship with pyridostigmine intake, and improvement following treatment [3]. Excessive acetylcholine causes parasympathetic overactivity and increased airway secretions, while nicotinic effects contribute to respiratory muscle paralysis. Similar clinical patterns have been shown in reports of distigmine, emphasizing that respiratory deterioration results from a combination of decreased secretion clearance and reduced respiratory muscle strength [11,12].

Cholinergic crisis results from acetylcholinesterase inhibition, leading to the excessive accumulation of acetylcholine at synapses and overstimulation of both muscarinic and nicotinic receptors [10-12]. Muscarinic toxicity manifests as parasympathetic overactivity (e.g., miosis, diaphoresis, lacrimation, salivation, and increased airway secretions). Conversely, nicotinic toxicity exerts effects on skeletal muscles such as respiratory muscle paralysis, tremors, and fasciculations, which can contribute to ventilatory failure [11,12]. In severe cases, this can progress to altered consciousness, seizures, and cardiopulmonary collapse accompanied by respiratory muscle paralysis [11].

Mechanistically, distigmine is characterized by a long-lasting acetylcholinesterase inhibitory effect and drug accumulation, which is thought to be the reason why distigmine-related crises are reported more frequently than those related to pyridostigmine [17]. Furthermore, reports related to distigmine suggest that insufficient intake or fasting may increase drug exposure and trigger a crisis. This supports the concept that a vulnerable host state may lower the threshold for developing a crisis in the presence of acetylcholinesterase inhibition [10].

As a diagnostic aid, a marked decrease in serum ChE is a significant finding; however, ChE alone shows substantial inter-individual variability and does not necessarily correlate with clinical severity. Because serum albumin (Alb) is generally less variable than ChE, the cholinesterase-to-albumin ratio has been proposed as an adjunctive index to reduce the influence of baseline differences [18]. In the referenced study, receiver operating characteristic analysis suggested that a cutoff value of 25.0 provided high diagnostic performance (reported sensitivity 90% and specificity 89%, with high discriminatory metrics) [18]. In our case, the ChE/Alb ratio was markedly low at presentation (4.2) and was also below the proposed cutoff during a prior hospitalization (23.1), suggesting that longitudinal monitoring of ChE/Alb may provide an additional signal to prompt earlier reassessment of acetylcholinesterase inhibitor therapy in nutritionally vulnerable patients. In clinical practice, especially in hemodialysis patients with fragile nutritional status, this ratio should be considered an exploratory and auxiliary marker rather than a standalone diagnostic criterion.

The clinical message of this case is that pyridostigmine, which has been considered relatively safe with limited reports of toxicity, can cause a fatal cholinergic crisis in situations where renal impairment (dialysis) and frailty overlap. At the time of dialysis initiation, or when the progression of frailty becomes evident (such as after a fracture, anorexia, weight loss, or hypoalbuminemia), prescriptions should not be continued reflexively but should be re-evaluated, including dose reduction and intensified monitoring. We present this case to alert clinicians that when encountering similar elderly dialysis patients, they should review the continuation of treatment by paying attention not only to renal impairment but also to the progression of frailty.

## Conclusions

Cholinergic crisis can occur even with standard doses of pyridostigmine in patients with end-stage renal disease receiving hemodialysis. Clinicians should consider dose reduction and close monitoring when prescribing to patients with advanced renal impairment. In elderly patients with significant weight loss and severe hypoalbuminemia, the vulnerability to severe cholinergic toxicity may increase due to reduced physiological reserve. Early recognition of cholinergic signs, prompt discontinuation of the drug, and appropriate supportive therapy are essential.

## References

[1] Gilhus NE, Andersen H, Andersen LK (2024). Generalized myasthenia gravis with acetylcholine receptor antibodies: a guidance for treatment. Eur J Neurol.

[2] Farrugia ME, Goodfellow JA (2020). A practical approach to managing patients with myasthenia gravis-opinions and a review of the literature. Front Neurol.

[3] Adeyinka A, Patel A, Kondamudi NP (2025). Cholinergic crisis. StatPearls [Internet].

[4] Cronnelly R, Stanski DR, Miller RD, Sheiner LB (1980). Pyridostigmine kinetics with and without renal function. Clin Pharmacol Ther.

[5] (2026). U.S. Food and Drug Administration. Pyridostigmine bromide tablets, USP: prescribing information. https://www.accessdata.fda.gov/drugsatfda_docs/label/2024/020414Orig1s011corrected_lbl.pdf.

[6] Tseng HK, Cheng YJ, Yu HK, Chou KT, Pang CY, Hu GC (2025). Malnutrition and frailty are associated with a higher risk of prolonged hospitalization and mortality in hospitalized older adults. Nutrients.

[7] Zhang L, Yang P, Yin F, Zhang J, Zhao B, Zhou J (2024). Association between frailty and hypoproteinaemia in older patients: meta-analysis and systematic review. BMC Geriatr.

[8] Liu J, Feng X, Li M, Zhao T (2016). A case report of cholinergic crisis evolved from myasthenia gravis due to the tumor in trigone of bladder. Neuro Endocrinol Lett.

[9] Shinya H, Hakoda S, Kiuchi S (2009). An aged patient with dementia of cholinergic crisis by distigmine bromide. J Jpn Soc Emerg Med.

[10] Sato S, Yamashiro E, Kawamura S (2015). Distigmine- and rivastigmine-associated cholinergic crisis; fasting and pharmacokinetic considerations. J Jpn Soc Emerg Med.

[11] (2011). Highlight: Cholinergic crisis due to distigmine bromide. J Jpn Soc Intensive Care Med.

[12] Onodera M (2008). Acute poisoning of distigmine bromide. Chudoku Kenkyu.

[13] Eiermann B, Sommer N, Winne D, Schumm F, Maier U, Breyer-Pfaff U (1993). Renal clearance of pyridostigmine in myasthenic patients and volunteers under the influence of ranitidine and pirenzepine. Xenobiotica.

[14] (2026). National Center for Biotechnology Information (NCBI). PubChem compound summary for pyridostigmine (CID. https://pubchem.ncbi.nlm.nih.gov/compound/Pyridostigmine.

[15] (2022). CKN. Dialysis of drugs. https://www.ckn.org.au/system/files/2022%20Dialysis%20of%20Drugs%20Interative%20Final%20022222.pdf.

[16] Jang SM, Infante S, Abdi Pour A (2020). Drug dosing considerations in critically ill patients receiving continuous renal replacement therapy. Pharmacy (Basel).

[17] Obara K, Tanaka Y (2020). Sustainable effects of distigmine bromide on urinary bladder contractile function. Pharmacology.

[18] Kariyone K, Shimatani Y, Kurihara T, Nagao T, Fujita Y, Uesugi M (2010). Establishing indicators for diagnosis of cholinergic crisis [Article in Japanese]. Rinsho Byori.

